# Promotion of Exercise and Health for Older People in Primary Care: A Qualitative Study on the Potential, Experiences and Strategies of General Practitioners in Germany

**DOI:** 10.1007/s10935-023-00730-6

**Published:** 2023-03-30

**Authors:** Julian Wangler, Michael Jansky

**Affiliations:** Centre for General Medicine and Geriatrics, University Medical Center of the Johannes Gutenberg University Mainz, Am Pulverturm 13, 55131 Mainz, Germany

**Keywords:** Exercise, Health promotion, Prevention, Sports, Course offers

## Abstract

In advanced age, physical activity becomes an important element in maintaining one’s individual health. GPs are considered to be well suited for advising and attending to older patients according to the principles of (preventive) healthcare. The subject was examined in the context of a study that determined options for action, experiences and strategies relating to the physical activation of older patients by GPs. Between 2021 and 2022, 76 semi-standardised interviews amongst GPs were conducted in all federal states of Germany. The data were evaluated by qualitative content analysis. The category system includes: importance of promoting physical activity; focus of exercise counselling; procedure of counselling; overview of exercise offers and cooperation with healthcare stakeholders; challenges and optimisation approaches. Many interviewees were aware of the significance of promoting health and exercise among older persons. Some physicians paid attention to identifying suitable activities for patients and motivating them to participate on a long-term basis. Cooperations with local health stakeholders have been identified. The interviewees recognised various challenges, which were mainly attributable to the lack of structures for health promotion. Several of the GPs lacked an overview of the physical activity programmes. GPs should assume an active role in exercise and health promotion for older patients. For them to be able to refer their patients effectively to exercise opportunities offered, it will be of importance to integrate the GP setting into a community-based network of prevention. Training measures could support the GP team to refer to the value of physical activity and address need-based recommendations.

**Supplementary Information:**

The online version contains supplementary material available at 10.1007/s10935-023-00730-6.

## Introduction

In an advanced age, regular exercise becomes an important key element in long-term healthcare, as it strengthens the musculature, physical balancing and the cardiovascular system and minimises risk factors (Becker et al., 2007; Cardona et al., 2022). In addition, physical and mental well-being may benefit from a physically active everyday life and dementia diseases (Cardona et al., 2022; Cunningham & O’Sullivan, 2021; Dapp et al., 2009; Heath et al., 2012).

According to the National Recommendations for Exercise (developed and published by the Federal Ministry of Health in Germany), it is suggested that adults over 65 years of age are to engage in moderate-intensity aerobic physical activity at least 150 min per week or with higher intensity 75 min per week (Pfeifer & Rütten, 2017). Exercise forms recommended are, for example, walking, cycling or swimming. In addition, moderate strength training and support activities for stabilising flexibility and balance should also take place (BMG, 2018; Malik et al., 2014).

As they often know their patients many years, general practitioners are regarded as well suited exercise and health consultants particularly for persons of older age (Becker et al., 2007; Bücker et al., 2017; Füzéki et al., 2020; Kleinert et al., 2015; Krüger-Band, 2011; Tulloch et al., 2006). Accordingly, GPs can motivate health-promoting behavior through their good and continuous knowledge of their patients. They have a holistic view and understanding of their patients and are therefore able to identify needs and requirements. GPs are usually well networked at their location, as they are considered 'guides in the healthcare system' to other care actors. In this way, patients can theoretically be referred to these actors in a needs-based manner (Kleinert et al., 2015; Krüger-Band, 2011; Tulloch et al., 2006). GPs often not only have the opportunity to express their recommendations and referrals, but are also able to prescribe specific physical measures, e.g. functional training and, since 2012, a so-called ‘Prescription for Exercise’ which is also to serve the purpose of creating patient awareness (Löllgen et al., 2013; Vogt et al., 2019). International studies have shown that numerous GPs make use of their advisory function in the field of prevention and health promotion and advocate this, however, it is seldom based on a systematic approach, for which reason the individual medical procedure becomes crucial (Curbach et al., 2018; Füzéki et al., 2020; Hébert et al., 2012).

As yet, there are only few publications especially in the German-speaking counties that are concerned with the question, which role GPs are to assume when it comes to the advocacy and prescription of exercise (Cunningham & O’Sullivan, 2021; Gabrys et al., 2016; Prüfer et al., 2015). It is widely unknown whether counselling is based on a concept or on behavioural modification therapy, or to what extent physicians apply motivation techniques, how they define “physical activity”, for which patients they deem counselling to be indicated, and how often aftercare shall proceed (Füzéki et al., 2020).

According to older publications, registered physicians of various specialty groups may be convinced that many diseases of civilisation can be significantly influenced by exercise, however, occasionally uncertainties have been expressed as far as health-oriented exercise counselling was concerned (Ruhrmann, 2014). Considering the efficacy of given exercise counselling, qualitative studies revealed that less than half of the physicians assumed that patients would have taken up a sports activity as a result (Bücker et al., 2017; Ruhrmann, 2014). However, patient surveys revealed that patients who had received such counselling from either their general practitioner or medical specialist had felt well guided to initiate a change of lifestyle (Gubisch et al., 2014).

Individual data prove that the prescription practice among GPs concerning physical measures—for example, issuing a prescription for exercise—has as yet been comparably reluctant. This corresponds to problems like scarcity of time and resources (Tulloch et al., 2006; Ruhrmann, 2014) but also to challenges of motivating and activating patients in the right way (Leemrijse et al., 2015). Overall, there is no comprehensive determination of how GPs assess their options for action in relation to the promotion of physical activity and health in older people. In addition, findings on conditions and barriers of exercise counselling are rather lacking (Wangler & Jansky, 2020; Yarnall, 2003).

The present study intends to make a contribution towards exploring the status quo of the subject exercise promotion for older people in the GP setting. Research-guiding issues concerned the potentials GPs perceive in contributing to exercise promotion for people over 65 years of age, the procedures they consider promising, and the challenges they experience in connection with the subject. In this study, the understanding of the term ‘exercise promotion’ includes, in a very fundamental sense, the commitment and involvement of GPs with regard to the topic of exercise counseling for older people and to what extent they act as advisors on this topic. As a consequence, this can include general recommendations, but under certain circumstances also the specific prescription.

However, the study is not concerned with a systematic recording of prescriptions and referrals in the field of exercise or physical activation, but rather with basic attitudes, perceived options for action, experiences, patterns of behavior and strategies of GPs towards the subject at hand.

## Methods

### Study Design

The study is based on qualitative, semi-standardised guideline interviews. Against the background of the findings, approaches to strengthening the primary care of GPs were to be concluded.

### Interview Guidelines

Parallel to the research questions, we created the interview guidelines comprising 24 core questions (cf. online Appendix). This instrument was primarily derived from a literature survey (especially the expertise of Kleinert et al. [2015]) and a previous study (Wangler & Jansky, 2020).

Above all, the preliminary study mentioned was the central basis for development. As part of this study, primary care patients aged 65 and over were asked about their needs and experiences with regard to GP health and physical activity promotion. This exploratory interview study focused on the occurrence of GP exercise counselling and the importance of advice and reference to exercise offers. The results showed the great importance of the GP advice, but also deficits. This study offered the opportunity to reflect and adapt many questions for the GP perspective.

### Recruitment

Because of a qualitative-explorative procedure a limited number of offices were recruited systematically in all federal states of Germany.

First, a pool of 448 potential contact addresses was set up, using doctor finder search engines made available by the Associations of Statutory Health Insurance Physicians (Kassenärztliche Vereinigungen) of the individual federal states. The pool included a wide range of GPs’ offices across all of Germany’s 16 federal states. We then began recruiting the sample.

106 GPs from the pool of contact addresses were contacted via telephone or e-mail. These doctors were selected on the basis of various criteria that were intended to ensure that a broad spectrum of GPs is represented in the sample (among other things gender, form of establishment, office environment [rural/urban], further education background). The aim was to have each federal state nearly equally represented in the study regardless of the number of inhabitants. In addition, value was placed on the geographical distribution of the medical offices within the federal states to be as broad as possible and to consider various age groups along with further qualification backgrounds. This way we were able to gain a heterogeneous sample.

In total, from the 106 GPs contacted, we conducted 76 interviews. While 5 GPs were recruited from each of 14 federal states, this figure was 3 for the federal states of Bremen and Saarland. The GPs all work in different practices or at different locations and take on tasks within the framework of statutory health insurance.

### Implementation and Sample

The interviewees received advance information about the subject of the interview as well as a written informed consent document. The interviews were held in the time between September 2021 and April 2022 and in 34 out of 76 cases were conducted by telephone (40 to 75 min). Theoretical saturation was reached.

Table 1 shows the gained sample.

**Table 1:** Sociodemographics of the survey sample rate (N = 76)

Age	Ø 54 years
Gender/sex	38 male, 38 female
Form of establishment	41 single medical office owners, 35 joint medical offices
Office environment	31 Country community/small town, 31 medium town, 14 big city
Status	55 office owners, 21 employed physicians
Further qualification background	19 subject-related further qualifications, 11 sports medical, 8 geriatric further qualifications

### Data Analysis

The first author evaluated the transcripts prepared after data collection using qualitative content analysis according to Mayring (2010, MAXQDA software, version 2020). This first entailed pinpointing the key statements, which was followed by further abstraction and summarisation, finally leading to a categorised system closely based on the interview guidelines and repeatedly reviewed and modified as necessary during evaluation. Our focus lay on forming categories from the various opinions and experiences. The category system created includes the following categories (subcategories in brackets):

- Importance of promoting physical activity in older age (importance of GP‘s advice; GP self-image towards to exercise and sport in patient care; Perceived potential for action of the GP as a (primary) contact person in matters of physical activity and sports; own practice situation: Importance of promoting health and physical activity in older people)
- Reason and focus of exercise counselling (occurrence of physical activity counselling; perception of demands and needs for physical activity promotion in older patients; type and content of exercise counseling; collaboration with and referral to other healthcare stakeholders; use of information material for exercise advice);
- Procedure of exercise counselling (specifics of older patients when it comes to exercise and sport; experiences with physical activity counseling for older people; success and observed effects of exercise counseling; approaches or strategies to contribute to the success of exercise counseling)
- Overview of local exercise offers and cooperation with healthcare stakeholders (knowledge and (subjectively felt) overview regarding offers for physical activity and health promotion for older patients; cooperation with other health stakeholders on the topic of physical activity promotion for older patients; evaluation of the cooperation with specialists with regard to the topic of physical activity promotion)
- Challenges and optimisation approaches (perceived challenges and problems in the field of health and physical activity promotion; willingness to give patients more support in the field of health and exercise promotion; improvement approaches for a stronger GP role in the field of health and physical activity promotion)

Theoretical saturation became apparent after 62 interviews. However, we had set the prior condition that all 76 interviews were to be conducted.

## Results

### Importance of Promoting Physical Activity in Older Age

Nearly all interviewees (72) considered exercise and health promotion as being a (very) important task field of (primary) care. This applied particularly to the group of older patients to whom the value of fitness and sports not only consists in exclusive healthcare. Instead, it cannot be esteemed high enough for their “subjective well-being […], for strengthening daily living skills and their feeling of still being able to do things […]” (I-22f).

Despite their emphasis on the subject of exercise, more than half of the interviewees (42) referred to the fact that everyday office routine would often not allow them to “manage a full, long-term consultations” (I-38 m). This would be particularly challenging in case of older people, as more specifics (e.g. previous diseases, compatibility with ongoing therapies) must be taken into consideration and the need for care would be greater. About one-third of the GPs (28) reported that they would advise elderly patients more often with regard to physical exercise; for one-third (26), this is occasionally the case.

When asked about their basic principles, the GPs interviewed perceived that they had various possibilities how to contribute to promoting physical activity:

In my opinion, sensitisation plays a central role, particularly in case of patients with pre-existing diseases. You must be familiar with these patients and you must be able to reach them with the necessary sensitivity (I-28m)

Of course, you can become active as a coach yourself, by agreeing with the patient on specific targets. But this requires time reserves and follow-up procedures (I-38m)

As GPs we are not unjustly referred to as being pilots in these systems. Here, I also perceive our duty in exercise and health promotion: we are mediators of various service offers. We give patients a profound overview and make recommendations. (I-34f)

### Reason and Focus of Exercise Counselling

The GPs we interviewed reported from experience that the starting points of exercise counselling for older patients would vary strongly.

The spectrum is broad. We have had older patients who are dissatisfied with their physical constitution and address us for this reason. But there is naturally also the group of chronically ill people who need special attention. Others in turn search for activities that keep them occupied and bring them in contact with other people. Especially as far as older patients are concerned, the reasons for wanting to be physically active cannot be generalised. (I-64m)

A larger fraction of the interviewed GPs (50) estimated that in most cases the subject of exercise and sports is initially addressed by themselves and counselling ensues on this basis. This applies in particular if exercise—prescribed as a longer term measure—promises a stabilisation or improvement of the patient’s health condition (e.g. obesity, diabetes).

Many GPs (60) held the opinion that “rather many” older people are willing to exercise on a regular basis (I-30f). However, they are often “unsure as to what extent it could have negative impact on certain ailments “ (I-54f), or to what extent sports programmes could exacerbate pre-existing maladies. Consequently, there would be an important need of this patient clientele “to get a kind of sense of security” (I-68f). Based on experience, the interviewees’ advice is taken seriously by most patients and submitted recommendations are accepted.

The majority of GPs reported that the forms of exercise they proposed to elderly patients primarily consisted of activities such as swimming, cycling, walking, (moderate) jogging or senior-friendly gymnastics and fitness courses.

The ideal exercise for seniors should focus on a combination of endurance, muscular strength and mobility (I-10m)

Important are light and comprehensive types of exercise (I-68f)

Most GPs interviewed (52) limited themselves to giving general advise or recommendations; a smaller fraction (22) wrote out exercise plans in individual cases and defined goals to be achieved together with the patient. Here, specific, partially reimbursable measures were prescribed, for example, consisting in functional training or rehabilitation sports. Common was the support of the interviewees’ recommendations with aids such as brochures and information leaflets (sports for senior citizens, less often community-based opportunities) or reference to health pages on the internet. Some of the interviewees (8), e.g. especially physicians who had been trained in sports medicine, were active in healthcare networks and, referred their patients to selected health and sports centres and physiotherapists, as needed and if patients were interested.

### Procedure of Exercise Counselling

The interviewees articulated that it would be important to create an understanding for the benefits of exercise among older patients, not just through education, but also by addressing the already mentioned “need for safety” (I-68f) by giving perfectly appropriate recommendations and advising their patients not to take any risks when engaging in physical activities.

In general, you will be more careful as an older person. That is why the elderly choose to rely on professional guidance. They are looking for an opportunity to approach an exercise offer that suits them and gradually feel their way in, preferably without risks. (I-35f)

It would also be of central importance not just to appeal to the patient to do exercises, but rather encourage an “intrinsic motivation” in him or her, which can be “internalised quickly in the sense of a natural daily or weekly rhythm” (I-40 m).

Sports are supposed to be fun. Naturally, I also try to communicate that it can also imply a social activity. You have to help the patient to understand that he or she will benefit from this everyday activity on very different fronts (I-76m)

You will have done right, if the patient does not perceive exercise as something imposed (I-32f)

It is advocated that upon suggesting certain activities the general practitioner shall anticipate “how well the chosen activity would be compatible with the patient’s personality” (I-40 m) and how good it could be “integrated into the patient’s everyday life and living conditions” (I-72f).

It would be of central importance “to have a clear awareness as to what extent an exercise activity to be recommended or prescribed shall produce purely physical effects, or whether it is also about strengthening psychosocial resilience” (I-10 m).

Elderly patients are sometimes affected by depressive phases. These can be well encountered with physical exercises . […] In some patients, the question of physical fitness is not even a priority (I-56m)

Many interviewees also underscore the importance of a realistic applicability of the recommendations. The physical exercise options must therefore be perceived as “low threshold” as possible (I-64 m).

In cases in which exercise counselling has taken place, a greater proportion of the sample described positive experiences and effects. It has been observed that upon starting to engage in exercise activities older patients “developed a routine perhaps quicker and more sustainably” than younger ones and felt well in doing so (I-68f).

### Challenges and Optimisation Approaches

However, it is felt to be problem that exercise counselling “by far cannot be done wherever it would be useful” (I-38 m).

According to my experience, it is not even so that patients would be so demotivated or that physical activity would do much good. No, the problem is rather that the general framework conditions are insufficient. (I-34f)

On the one hand, this is justified by a lack of time, on the other, by the absence of adequate structures and contact points for the promotion of physical activity.

Practically, I stand all alone with this. Where are the helping hands? Where are the systematic support programmes to which I can refer my patients? Where can I get an overview and information? Where are higher-level alliances? (I-44m)

Apart from any sports initiatives, which perhaps might exist here and there on the community level I perceive no comprehensive network, in which we as GPs are able to participate. As in so many other fields, we are lone fighters. It deprives us of many opportunities (I-54f)

From the perspective of many GPs, this is accompanied by an unsatisfactory remuneration situation.

In my opinion, we have a real incentive problem on the remuneration side. There is no possibility of correctly billing consulting services in the area of physical activity promotion. Health policy has to consider this: If we as GPs are to enter this field of action, we must also be able to bill services better. (I-12m)

Several GPs criticised that they would feel “let down a little” when it comes to issues of health promotion (I-2 m) and, left to their own devices, they would not have a sufficient overview of local sports offers and could not refer their patients to them quickly and free of complications.

Both transparency and contact persons are needed, particularly when it is about physical fitness in old age. […] I see that the communities and the federal states have a duty to do more. They have to build a network-like, interdisciplinary structure in which many actors come together. (I-44m)

One part of the interviewed GPs were familiar with prescription options such as the issuance of a prescription for exercise (42) or with the *SPORT PRO GESUNDHEIT* courses subsidised by the health insurances (36). According to their own statements, a number of physicians (32) were “relatively reluctant” to prescribe functional training (I-28 m). As a reason for this, one part of the interviewees admitted that they “did not feel fully knowledgeable” about exercise counselling and prescriptions (I-70 m) or “did not always take prescriptions into consideration right away” (I-46f). Some GPs also did not consider themselves as primarily responsible for prescribing physical activity and rather referred their patients to medical specialists or physiotherapists.

I see myself more as a guide in the system on this issue. That means I see myself as an effective referrer to the specialists who are able to work well with patients on such issues of prevention and health promotion. (I-27m)

With a view to possible optimisation approaches, the GPs interviewed articulated, apart from their longing for appropriate structures and programmes, “a broad range of attractive further qualification formats for physicians and medical office staff” (I-58f). Possibilities of delegation were envisaged particularly with respect to the latter issue.

The possibilities of delegation are surely not exhausted yet. Further qualified office staff members are also able to support, inform, and motivate patients, for which the general practitioner is not necessarily needed. (I-58f)

Some interviewees saw opportunities in health apps and the federal government's recent ability to prescribe licensed digital health apps (DiGA). The potentials of health apps were seen particularly in the areas of motivation, information and life style change. The playful element in particular was considered to be highly useful in promoting exercise and maintaining health among older people.

Well designed, serious health apps can certainly make a contribution as one element in an overall concept to implement exercise lastingly in everyday life and increase the fun factor. (I-54f)

It has also been suggested that the health insurance companies should make their insured elderly members more aware of preventive services. This would be a contribution to relieve the physicians from having to search and find suitable offers.

Think of the resources and knowledge that organizations such as statutory health insurance companies have. They also have access to patients. Patients could be informed more quickly and effectively via health insurance. (I-44m)

## Discussion

In the course of the interview study, the awareness and attitude of a mixed sample of GPs towards exercise promotion for older patients became visible. The existing networks, behavioural patterns as well as promotion factors and challenges were synthesised by the interviews. In total, they revealed a distinctive degree of knowledge and sensitivity among the GPs for health and exercise promotion issues in old age. Many of the GPs interviewed showed great dedication when asked to identify suitable activities for older patients and motivate their clientele to take part in them in the long term. Some cooperated with local sports and health organisations to be able to refer to specific options and ascertain their appropriateness.

The interviewees also made mention of obstacles and challenges. For example, one part of the GPs perceived that there is a significant deficit due to the absence of adequate structures and programmes when it comes to exercise promotion. In analogy to this, the reimbursement system was criticised. In this regard, the interviewees refer to the reimbursement conditions of GP counselling services for health promotion by the creation of billing codes adjusted to the effort involved. In addition, a number of GPs lacked an overview of the available fitness and sports opportunities offered in their vicinity; cooperation with health providers does not always exist. Furthermore, one part of the GPs interviewed made only limited use of prescription possibilities such as functional training and was not familiar with prescribing exercises.

Many of these results are in agreement with the existing study situation, according to which the GP setting is well suited to advise elderly patients in matters of health-promoting exercise and motivate them to take advantage of individually suitable exercise offers in the longer term (Ruhrmann, 2014). GPs already have a variety of options available in order to promote exercise and health as, for example functional training or rehabilitation sports. Occasions such as preventive medical check-ups can be systematically used to refer to the value of continuous physical activity and to express recommendations (Gabrys et al., 2016; Kleinert et al., 2015).

At the sime time, the results of the present study reveal problems such as a scarcity of time and resources (Tulloch et al., 2006;) as well as a deficiency of financial reimbursements (Wangler & Jansky, 2020; Yarnall, 2003). A survey in which 800 Dutch GPs were questioned with regard to the subject of promotion of physical activity showed that addressing, motivating and activating patients in the right way also represents a significant barrier for doctors (Leemrijse et al., 2015). There were also difficulties because special counselling and treatment solutions were either not widely known among GPs or were being applied to a limited degree only (Gubisch et al., 2014; Till et al., 2022). As a result, referral to local exercise facilities is low in primary care (Leemrijse et al., 2015). In addition, it would also be difficult to keep track of or gain access to local courses and support options, the same also applied to advice material (Tulloch et al., 2006). Earlier studies revealed that there had occasionally been uncertainties concerning health-oriented exercise counselling on the part of the physicians (Braumann et al., 2001). Nevertheless, a survey conducted among patients who received counselling elicited that the medical support had been assessed as being (very) helpful (Gubisch et al., 2014).

In a survey of older patients, the problem was shown to be that the patients were often interested in exercise offers, but not seldom lacked a competent, trustworthy agent able to mediate these offers as needed (Wangler & Jansky, 2020). According to the interviewees, GPs enjoyed great trust as mediators in matters of health promotion. However, only about 40% reported that their general practitioner had ever recommended them specific health services. However, whenever these recommendations had been communicated most of the interviewees not only tested the respective offer, but also took advantage of it for a longer period of time.

A number of publications encouraged to make more consistent use of the GP setting in order to contribute to encourage physical activities of older people (Gabrys et al., 2016; Peters et al., 2017). However, GPs often lack the time and resources. For this reason, concepts have already emerged that try to anchor health promotion more firmly in primary care without unnecessarily burdening GPs. A key to this could be delegation and further training approaches for practice staff and special case managers, who could be a transmission belt towards exercise providers (Füzéki et al., 2020). Targeted training measures as well as further qualification formats should be made available to GP practices so that the GP team can use the office visits of elderly patients systematically to refer to the value of physical activity, passing on information and addressing specific recommendations. First concepts on this subject have already been submitted and evaluated (Bücker et al., 2017; Intorp, 2015; Peters et al., 2017; Till et al., 2022).

Health policy support for GPs is also a possible starting point. Exemplary in this regard is the study of Prüfer et al. (2015) who saw great potential in embedding GP prevention work into a local/community system of health promotion using formal and informal networks to reinforce health promotion. Accordingly, the inclusion and coordination of various health promotion actors is the key to achieve a more effective GP care and a coordination of exercise and health promotion tasks (Cunningham & O’Sullivan, 2021; Leemrijse et al., 2015).

When it comes to accomplishing needs-based healthcare provisions for older people, a cooperation between GPs and other healthcare professions (physiotherapists, coaches) will be an advantage. Prüfer et al. have advocated a modification of structural framework conditions, in particular a reduction of cooperation barriers and a precise definition of the roles of the actors involved as well as the provision of sufficient resources.

The targeted application of digital tools to support continual physical exercise in advanced age can also add value to primary care (Fischer et al., 2020). As patient surveys have already shown, patients not only want their general practitioner to assume a moderating role in the mediation of exercise offers, but also wish to get more support from their health insurance companies (Vogt et al., 2019).

Although a heterogeneous sample was recruited, the study has several limitations. Apart from the limited number of respondents and the qualitative approach, it has a focus on regional recruitment.

As far as the cooperation willingness of the interview partners is concerned, it may be considered that more GPs who are interested in the subject could have participated. Besides, a large proportion of the interviews were conducted by telephone which, as compared with face-to-face interviews, might have had an influence on the significance of the results.

The survey was exclusively focused on exercise promotion. This dimension is often associated with other topics in the reality of healthcare provision, e.g. lifestyle and nutrition. Such interactions have not been examined here.

The Interviews revealed that many GPs were aware of the significance of promoting health and exercise among older persons and basically consider the GP setting to be qualified in this field. Some physicians paid special attention to identifying suitable activities for older patients and motivating them to participate on a long-term basis. Various cooperation forms including local health and sports stakeholders have been identified. The interviewees recognised various challenges and problems in promoting physical activities among older patients, which were mainly attributable to the lack of structures and established programmes for physical activity and health promotion. Some of the GPs lacked an overview of the physical activity programmes that were being offered in their respective quarters; cooperation or contact with healthcare providers did not always exist.

It seems reasonable to encourage GPs to assume an active role in exercise and health promotion, in particular to the benefit of older patients. For them to be able to refer their patients effectively to exercise opportunities offered in their neighbourhoods, it can be helpful to integrate the GP setting into a community-based network of prevention and health promotion. Targeted training measures could support the GP team to use the office visits of older patients systematically to refer to the value of physical activity, pass on information and address need-based recommendations.

## Supplementary Information

Below is the link to the electronic supplementary material.

Supplementary file1 (PDF 302 kb)

## Data Availability

Data from this research are not publicly available because participants did not give permission for recordings or transcripts to be released to other researchers.

## Abbreviation

GP: General practitioner
