# Supplementary material for: Promotion of Exercise and Health for Older People in Primary Care: A Qualitative Study on the Potential, Experiences and Strategies of General Practitioners in Germany
Source: J Prev (2022). 2023 Mar 30;44(4):477–90. doi: 10.1007/s10935-023-00730-6 (PMC10423120; doi:10.1007/s10935-023-00730-6)
Supplement: Supplementary file 1 — Supplementary file1 (PDF 302 kb) [file 10935_2023_730_MOESM1_ESM.pdf]

**Interview guidelines**

*Importance of promoting physical activity in old age*

- What importance do you attach to the topic of exercise and sports in patient care?
- What role does a general practitioner have when it comes to the subject of promoting health and physical activity? To what extent do you perceive the general practitioner as a primary or important contact person in matters of physical activity and sports? To what extent is it desirable for a general practitioner to deal with this topic and advise his or her patients?
- What does your everyday practice look like: How important is promoting health and physical activity in this context?
- What possibilities and potentials do you as a general practitioner have in contributing to encourage physical activities in older people? (This patient group will be the focus of the following section.)
- How often do you advise older patients with regard to physical activity and sports?

*Reason, circumstances and focus of physical activity counselling*

- How does this counselling normally start? Do the patients come to you and ask for information/recommendations o do you usually address the topic of physical activity and/or sports on your own initiative?
- What are the genuine reasons? (prevention, pre-existing diseases and/or therapy measures , rehabilitation)
- How do you usually perceive the needs and requirements of exercise promotion among older patients? What significance does the general practitioner's advice have?
- What kind of physical activity do you recommend to older people?
- Do you exclusively provide general information on physical activities and give recommendations, or do you also define specific exercise plans/joint objectives together with your patient?
- To what extent do you prescribe specific physical measures or refer your patients to particular healthcare stakeholders? Why (not)?
- To what extent do you use aids such as brochures, information leaflets or recommend specific websites for physical activity counselling?

*Procedure of physical activity counselling , experiences with it and effects observed*

- What are specific characteristics apply to older patients when it comes to physical activity and sports? What should you as a general practitioner have to pay attention to when advising patients to exercise or concerning specific recommendations or prescriptions?

- What are your basic experiences with counselling older people? What kind of effects have you observed?
- According to your observations, how often does the physical activity counselling you provide bear fruit?
- In your experience, how well can older patients be motivated to engage in more sports and do more exercise? What works well, what less so? What does the long-term willingness of your patients to participate look like?
- Do you have specific procedures or strategies that might contribute to the successful outcome of exercise counselling?

*Overview of local exercise offers and cooperation with healthcare stakeholders*

- What opportunities of exercise and health promotion are available for older patients in your neighbourhood? To what extent would you say that you have a good overviews? How do you normally become aware of opportunities such as sports, fitness or exercise courses?
- To what extent do you cooperate in matters of promoting physical activities (for older patients) with other healthcare stakeholders (e.g. sports clubs, health centres, fitness studios, community physical activity initiatives, physiotherapists)? Why not? To what extent would a (stronger) cooperation be reasonable in this area?
- How do you judge the cooperation with medical specialists when it comes the subject of promoting physical activities?

*Experienced challenges and optimisation approaches*

- What challenges and problems do you perceive when it comes the subject of promoting physical activities of older people?
- According to your opinion, what needs to be done that you or (other) general physicians could render yet better support to older patients in the field of health and exercise promotion?
- It has been suggested that general practitioners should cooperate systematically with the suppliers of health and training courses (e.g. fitness studios, health insurance companies, adult education centres), if possible, perhaps allowing you to inform your patients quicker about opportunities or to refer them to such available services. What is your opinion?
- Should general practitioners assume a stronger role as mediators in the future, when it comes to referring (older) patients to healthcare services and exercise opportunities? Under what circumstances is this conceivable for you?
